# A bibliometric analysis of the top 100 most cited articles on corticospinal tract regeneration from 2004 to 2024

**DOI:** 10.3389/fnins.2024.1509850

**Published:** 2025-01-28

**Authors:** Saijilafu, Ling-Chen Ye, Huanyi Li, Haokun Li, Xinyi Lin, Kehui Hu, Zekai Huang, Chimedragchaa Chimedtseren, Linjun Fang, Saijilahu, Ren-Jie Xu

**Affiliations:** ^1^Hangzhou Lin’an Traditional Chinese Medicine Hospital, Affiliated Hospital, Hangzhou City University, Hangzhou, China; ^2^Key Laboratory of Novel Targets and Drug Study for Neural Repair of Zhejiang Province, School of Medicine, Hangzhou City University, Hangzhou, China; ^3^Department of Orthopaedics, Suzhou Municipal Hospital, The Affiliated Suzhou Hospital of Nanjing Medical University, Suzhou, China; ^4^Institute of Traditional Medicine and Technology of Mongolia, Ulaanbaatar City, Mongolia; ^5^Tongliao Centers for Disease Control and Prevention, Tongliao, China

**Keywords:** corticospinal tract regeneration, bibliometric analysis, Web of Science Core Collection, VOSviewer, CiteSpace

## Abstract

**Objective:**

Here, bibliometric and visual analytical techniques were employed to assess the key features of the 100 most cited publications concerning corticospinal tract (CST) regeneration.

**Methods:**

Research was conducted within the Web of Science Core Collection to pinpoint the 100 most cited publications on CST regeneration. From these, comprehensive data encompassing titles, authorship, key terms, publication venues, release timelines, geographic origins, and institutional affiliations were extracted, followed by an in-depth bibliometric examination.

**Results:**

The 100 most cited publications were all published between 2004 and 2024. These seminal papers amassed an aggregate of 18,321 citations, with individual citation counts ranging from 83 to 871 and a median of 136 citations per paper. Schwab M. E., stood out as the most prominent contributor, with significant authorship in 9 of the 100 papers. The United States dominated the geographical distribution, accounting for 49 of the articles. With 17 publications, the University of California System led the institutional rankings. A thorough keyword analysis revealed pivotal themes in the field, encompassing the optic nerve, gene expression, CST integrity and regeneration, diffusion tensor imaging, myelin-associated glycoproteins, inhibitors of neurite outgrowth, and methods of electrical and intracortical microstimulation.

**Conclusion:**

This investigation provides a bibliometric analysis of CST regeneration, underscoring the significant contribution of the United States to this field. Our findings unveiled the dynamics and trends within the field of CST regeneration, providing a scientific foundation for advancing clinical applications. Building on this analysis, the clinical application of CST regeneration should be optimized through interdisciplinary collaboration, enabling the exploration and validation of a variety of therapeutic approaches, including the use of neurotrophic factors, stem cell therapies, biomaterials, and electrical stimulation. Concurrently, additional clinical trials are necessary to test the safety and efficacy of these therapeutic methods and develop assessment tools for monitoring the recovery of patients. Furthermore, rehabilitation strategies should be refined, and professional education and training should be provided to enhance the understanding of CST regeneration treatments among both medical professionals and patients. The implementation of these strategies promises to enhance therapeutic outcomes and the quality of life of patients with spinal cord injury (SCI).

## 1 Introduction

The corticospinal tract (CST) is a descending motor pathway that originates in the cerebral cortex and projects to the spinal cord. It plays a crucial role in controlling voluntary movements, fine motor coordination, and executive functions (Welniarz et al., 2017). The integrity of the CST is essential for the precise execution of skilled motor activities. Damage to it can result in significant motor impairments, as seen in conditions such as spinal cord injury (SCI), multiple sclerosis, and stroke (Oudega and Perez, 2012).

Understanding the mechanisms involved in CST regeneration has been a key focus in neuroscientific research, aiming to unlock new therapies for patients with motor disabilities. The limited capacity of the CST for self-repair post-injury presents a significant challenge to regeneration. Accordingly, researchers have explored various strategies to enhance the regenerative potential of the CST, including the use of growth factors, cell transplantation, and the development of biomaterials to support axonal regrowth (Blesch et al., 2002; Zuo et al., 2020).

Bibliometric analysis is a powerful tool for identifying trends, patterns, and key contributors in CST regeneration research (Ellegaard and Wallin, 2015). This approach has previously been used to evaluate research across medical and scientific fields, offering insights into the structure and dynamics of scientific knowledge (Moed, 2009).

In this study, we conducted a bibliometric analysis of the 100 most cited articles on CST regeneration, providing a comprehensive overview of the advancements in this field. Our analysis spanned 20 years (2004 to 2024) using data from the Web of Science Core Collection, a leading repository for peer-reviewed literature. Through the examination of the characteristics of the most cited papers, we sought to identify the main research themes, methodologies, and the geographical distribution of scientific output in this field.

Our detailed bibliometric analysis offers insights into the scientific community’s efforts to advance knowledge and promote innovation in CST recovery.

## 2 Materials and methods

### 2.1 Search strategies and data extraction

**Figure 1:**
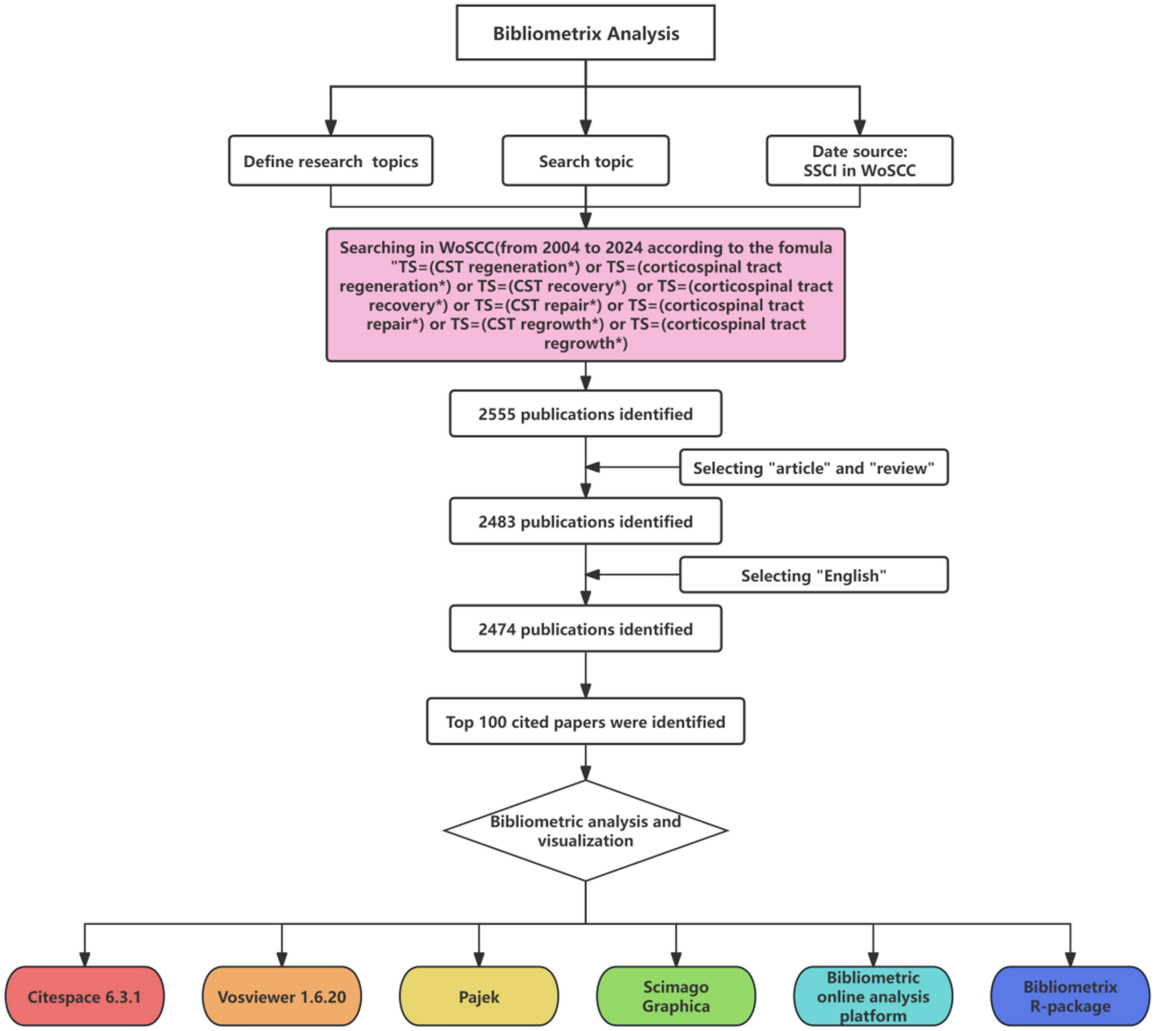
Document screening flow chart.

The data for this investigation were sourced from the Science Citation Index Expanded (SCI-EXPANDED), a component of the Clarivate Analytics Web of Science Core Collection (WoSCC). Recognized as a prestigious global resource, the WoSCC offers extensive data necessary for bibliometric analysis, making it a preferred database for such studies. The search criteria incorporated terms such as “CST regeneration,” “corticospinal tract regeneration,” “CST recovery,” “corticospinal tract recovery,” “CST repair,” “corticospinal tract repair,” “CST regrowth,” and “corticospinal tract regrowth” to identify pertinent articles. A systematic search covered publications from January 1900 to September 2024, with the retrieval process being finalized on September 16, 2024, ensuring that no post-update database changes would influence the results. The bibliometric analysis included only original articles and review papers with complete manuscripts, with the language limited to English. Two independent researchers conducted the initial screening based on titles, abstracts, and document types, with full-text reviews being conducted when necessary for a thorough assessment. The top 100 articles, ranked by their influence, were retrieved from WoSCC in “Full Record and Cited References” format and were saved as TXT files (Figure 1). The impact factors of the relevant journals were extracted from Journal Citation Reports 2023.

### 2.2 Data analysis and visualization

Descriptive statistical analysis and diagram creation were conducted using Microsoft Excel 2019. An online analytical platform was used to enhance the visualization of collaborative networks among authors and institutions. VOSviewer, a powerful bibliometric tool developed by van Eck and Waltman (2010), was employed for constructing networks of keywords and authors. Additionally, Scimago Graphica (Dimara and Perin, 2020) was used to generate a world map representing the cited countries. Pajek (Batagelj and Mrvar, 2004), a robust network analysis tool, was utilized for the visualization of intricate citation networks, co-word analysis, and author collaboration networks. Its integration with VOSviewer enabled a comprehensive perspective on the scientific literature network structure, highlighting major trends, key concepts, and collaborative ties among researchers, which is invaluable for understanding the broader academic research landscape. Furthermore, CiteSpace (Chen, 2004) software was used to map author networks, pinpoint keywords with significant burst strength, and showcase the temporal dynamics of keyword occurrences. The data collected in this investigation were compiled in CiteSpace to create a network of institutional collaborations, authorships, and co-occurrences. Within the CiteSpace network map, diverse entities, including institutions, authors, and keywords, are depicted through nodes, with the line thickness between nodes indicating the intensity of collaboration and co-occurrence. Co-occurrence analysis establishes item correlations based on their co-occurrence frequency within documents, color-coded to represent different clusters. Bibliometrix, an R package for bibliometric analysis, expedites the discovery of foundational works, influential authors, and emerging trends within a field. It also assists in curating and visually presenting research findings. A distinctive feature of CiteSpace Bibliometrix is its capacity to generate Sankey diagrams, visualizing data flows with branch widths corresponding to flow magnitudes, thus serving as an effective analytical tool.

## 3 Results

### 3.1 Analysis of publications and citations

**Figure 2:**
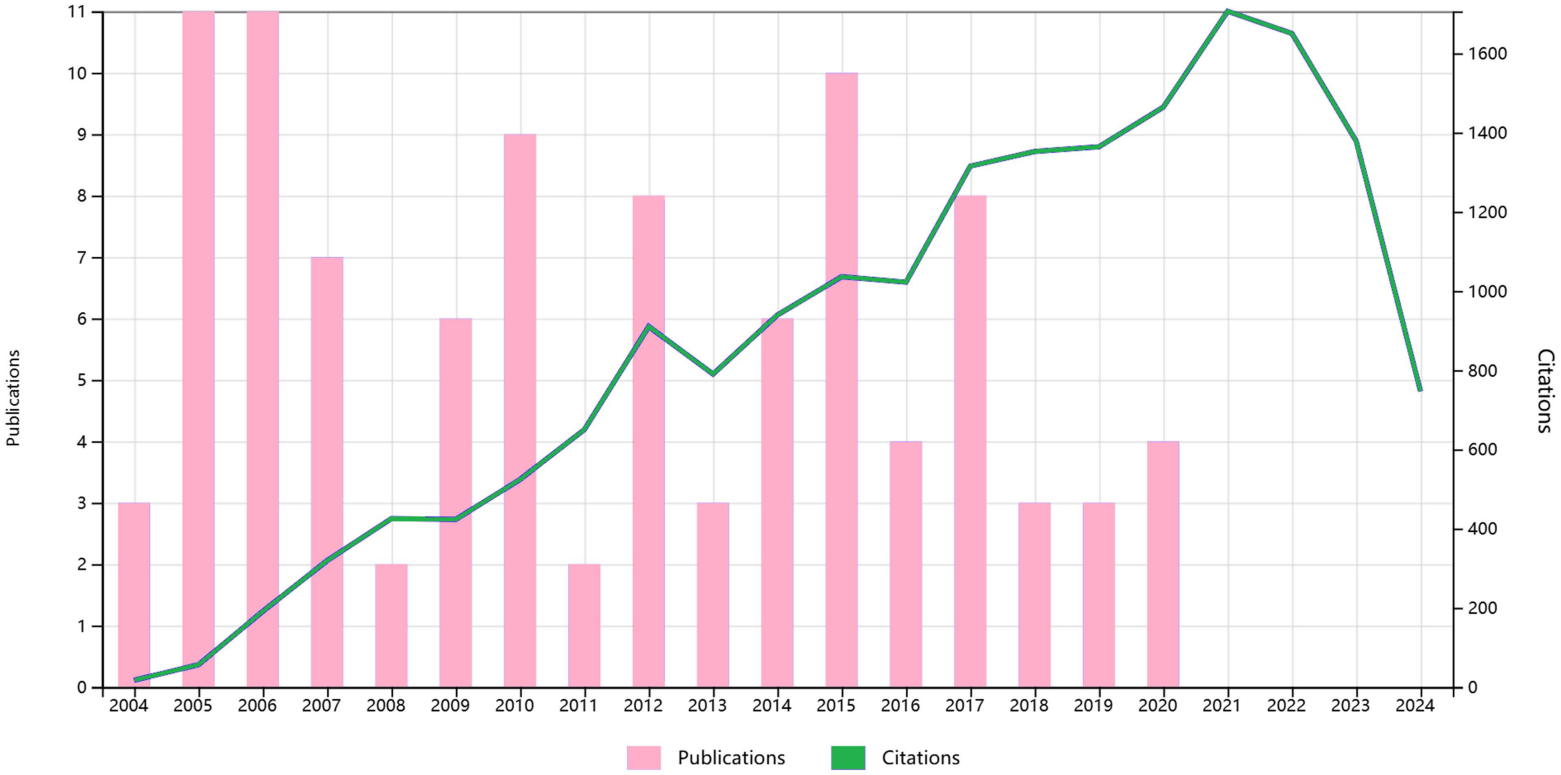
The annual number of publications and citations for the 100 most cited papers from 2004 to 2024.

The 100 most cited studies in this field were published between 2004 and 2020, as shown in Figure 2. Table 1 presents a detailed breakdown of these 100 publications. These leading 100 articles have amassed citations ranging from 83 to 871, with a median citation count of 136 and an average citation frequency of 183.21 per article. Of the top three most cited studies, the preeminent one, titled (Bareyre et al., 2004) “The injured spinal cord spontaneously forms a new intraspinal circuit in adult rats,” was published in Nature Neuroscience in 2004 and garnered 871 citations. Following closely in second was a study titled (Liu et al., 2010) “PTEN deletion enhances the regenerative ability of adult corticospinal neurons,” which appeared in Nature Neuroscience in 2010 and amassed 725 citations. The third study (Stinear et al., 2006), “Functional potential in chronic stroke patients depends on corticospinal tract integrity,” was published in Brain in 2007, and accumulated 637 citations. While earlier publications tended to have higher overall citation counts, a closer examination based on the average annual citations revealed several more recent publications with a markedly higher impact. Notably, the paper titled (Silva et al., 2014) “From basics to clinical: a comprehensive review on spinal cord injury” published in Progress in Neurobiology in 2014, secured 574 citations, placing it fourth in terms of total citations.

**Table 1:** The 100 most-cited articles in CST generation.

Number	First author	Publication year	Title	Journal	Total citations
1	Bareyre F. M.	2004	The injured spinal cord spontaneously forms a new intraspinal circuit in adult rats (Bareyre et al., 2004)	Nature Neuroscience	871
2	Liu K.	2010	PTEN deletion enhances the regenerative ability of adult corticospinal neurons (Liu et al., 2010)	Nature Neuroscience	725
3	Stinear C. M.	2007	Functional potential in chronic stroke patients depends on corticospinal tract integrity (Stinear et al., 2006)	Brain	637
4	Silva N. A.	2014	From basics to clinical: a comprehensive review on spinal cord injury (Silva et al., 2014)	Progress in Neurobiology	574
5	García-Alías G.	2009	Chondroitinase ABC treatment opens a window of opportunity for task-specific rehabilitation (García-Alías et al., 2009)	Nature Neuroscience	371
6	Dickendesher T. L.	2012	NgR1 and NgR3 are receptors for chondroitin sulfate proteoglycans (Dickendesher et al., 2012)	Nature Neuroscience	356
7	Barritt A. W.	2006	Chondroitinase ABC promotes sprouting of intact and injured spinal systems after spinal cord injury (Barritt et al., 2006)	Journal of Neuroscience	339
8	Liu K.	2011	Neuronal intrinsic mechanisms of axon regeneration (Liu et al., 2011)	Annual Review of Neuroscience	334
9	Kremenchutzky M.	2006	The natural history of multiple sclerosis: a geographically based study 9: observations on the progressive phase of the disease (Kremenchutzky et al., 2006)	Brain	331
10	Karimi-Abdolrezaee S.	2010	Synergistic effects of transplanted adult neural stem/progenitor cells, chondroitinase, and growth factors promote functional repair and plasticity of the chronically injured spinal cord (Karimi-Abdolrezaee et al., 2010)	Journal of Neuroscience	292
11	Hamers F. P. T.	2006	CatWalk-assisted gait analysis in the assessment of spinal cord injury (Hamers et al., 2006)	Journal of Neurotrauma	282
12	Kadoya K.	2016	Spinal cord reconstitution with homologous neural grafts enables robust corticospinal regeneration (Kadoya et al., 2016)	Nature Medicine	267
13	Byrnes K. R.	2005	Light promotes regeneration and functional recovery and alters the immune response after spinal cord injury (Byrnes et al., 2005)	Lasers in Surgery and Medicine	266
14	Freund P.	2006	Nogo-A-specific antibody treatment enhances sprouting and functional recovery after cervical lesion in adult primates (Freund et al., 2006)	Nature Medicine	257
15	Lo Giudice T.	2014	Hereditary spastic paraplegia: clinical-genetic characteristics and evolving molecular mechanisms (Lo Giudice et al., 2014)	Experimental Neurology	256
16	Wahl A. S.	2014	Asynchronous therapy restores motor control by rewiring of the rat corticospinal tract after stroke (Wahl et al., 2014)	Science	252
17	Zaaimi B.	2012	Changes in descending motor pathway connectivity after corticospinal tract lesion in macaque monkey (Zaaimi et al., 2012)	Brain	248
18	Lee J. K.	2010	Assessing spinal axon regeneration and sprouting in Nogo-, MAG-, and OMgp-deficient mice (Lee et al., 2010)	Neuron	240
19	Feng W.	2015	Corticospinal tract lesion load: an imaging biomarker for stroke motor outcomes (Feng et al., 2015)	Annals of Neurology	237
20	Zhu L. L.	2010	Lesion load of the corticospinal tract predicts motor impairment in chronic stroke (Zhu et al., 2010)	Stroke	237
21	Tuszynski M. H.	2012	Concepts and methods for the study of axonal regeneration in the CNS (Tuszynski and Steward, 2012)	Neuron	236
22	Dias D. O.	2018	Reducing pericyte-derived scarring promotes recovery after spinal cord injury (Dias et al., 2018)	Cell	234
23	Sasaki M.	2009	BDNF-hypersecreting human mesenchymal stem cells promote functional recovery, axonal sprouting, and protection of corticospinal neurons after spinal cord injury (Sasaki et al., 2009)	Journal of Neuroscience	233
24	Harel N. Y.	2006	Can regenerating axons recapitulate developmental guidance during recovery from spinal cord injury? (Harel and Strittmatter, 2006)	Nature Reviews Neuroscience	232
25	Hata K.	2006	RGMa inhibition promotes axonal growth and recovery after spinal cord injury (Hata et al., 2006)	Journal of Cell Biology	232
26	Keefe K. M.	2017	Targeting neurotrophins to specific populations of neurons: NGF, BDNF, and NT-3 and their relevance for treatment of spinal cord injury (Keefe et al., 2017)	International Journal of Molecular Sciences	226
27	Ramer L. M.	2014	Restoring function after spinal cord injury: towards clinical translation of experimental strategies (Ramer et al., 2014)	Lancet Neurology	225
28	Zheng B. H.	2005	Genetic deletion of the Nogo receptor does not reduce neurite inhibition or promote corticospinal tract regeneration *in vivo* (Zheng et al., 2005)	Proceedings of the National Academy of Sciences of the United States of America	225
29	Freund P.	2013	MRI investigation of the sensorimotor cortex and the corticospinal tract after acute spinal cord injury: a prospective longitudinal study (Freund et al., 2013)	Lancet Neurology	211
30	Zukor K.	2013	Short hairpin RNA against PTEN enhances regenerative growth of corticospinal tract axons after spinal cord injury (Zukor et al., 2013)	Journal of Neuroscience	209
31	Stinear C. M.	2017	Prediction of motor recovery after stroke: advances in biomarkers (Stinear, 2017)	Lancet Neurology	206
32	Blackmore M. G.	2012	Krüppel-like factor 7 engineered for transcriptional activation promotes axon regeneration in the adult corticospinal tract (Blackmore et al., 2012)	Proceedings of the National Academy of Sciences of the United States of America	202
33	Cafferty W. B. J.	2010	MAG and OMgp synergize with Nogo-A to restrict axonal growth and neurological recovery after spinal cord trauma (Cafferty et al., 2010)	Journal of Neuroscience	202
34	Girgis J.	2007	Reaching training in rats with spinal cord injury promotes plasticity and task specific recovery (Girgis et al., 2007)	Brain	195
35	Thomas S. L.	2005	Increases in corticospinal tract function by treadmill training after incomplete spinal cord injury (Thomas and Gorassini, 2005)	Journal of Neurophysiology	193
36	Hutson T. H.	2019	The translational landscape in spinal cord injury: focus on neuroplasticity and regeneration (Hutson and Di Giovanni, 2019)	Nature Reviews Neurology	189
37	Fujimoto Y.	2012	Treatment of a mouse model of spinal cord injury by transplantation of human induced pluripotent stem cell-derived long-term self-renewing neuroepithelial-like stem cells (Fujimoto et al., 2012)	Stem Cells	181
38	Piantino J.	2006	An injectable, biodegradable hydrogel for trophic factor delivery enhances axonal rewiring and improves performance after spinal cord injury (Piantino et al., 2006)	Experimental Neurology	169
39	Wang D.	2011	Chondroitinase combined with rehabilitation promotes recovery of forelimb function in rats with chronic spinal cord injury (Wang et al., 2011)	Journal of Neuroscience	159
40	Bunday K. L.	2012	Motor recovery after spinal cord injury enhanced by strengthening corticospinal synaptic transmission (Bunday and Perez, 2012)	Current Biology	158
41	Brus-Ramer M.	2007	Electrical stimulation of spared corticospinal axons augments connections with ipsilateral spinal motor circuits after injury (Brus-Ramer et al., 2007)	Journal of Neuroscience	157
42	Bareyre F. M.	2005	Transgenic labeling of the corticospinal tract for monitoring axonal responses to spinal cord injury (Bareyre et al., 2005)	Nature Medicine	156
43	Fischer I.	2020	Transplanting neural progenitor cells to restore connectivity after spinal cord injury (Fischer et al., 2020)	Nature Reviews Neuroscience	153
44	Liu Z.	2014	Beneficial effects of GFAP/vimentin reactive astrocytes for axonal remodeling and motor behavioral recovery in mice after stroke (Liu et al., 2014)	Glia	149
45	He M.	2016	Autophagy induction stabilizes microtubules and promotes axon regeneration after spinal cord injury (He et al., 2016)	Proceedings of the National Academy of Sciences of the United States of America	143
46	Jankowska E.	2006	How can corticospinal tract neurons contribute to ipsilateral movements? A question with implications for recovery of motor functions (Jankowska and Edgley, 2006)	Neuroscientist	143
47	Liu Y.	2017	A sensitized IGF1 treatment restores corticospinal axon-dependent functions (Liu et al., 2017)	Neuron	142
48	Poplawski G. H. D.	2020	Injured adult neurons regress to an embryonic transcriptional growth state (Poplawski et al., 2020)	Nature	138
49	Friedli L.	2015	Pronounced species divergence in corticospinal tract reorganization and functional recovery after lateralized spinal cord injury favors primates (Friedli et al., 2015)	Science Translational Medicine	137
50	Klapka N.	2005	Suppression of fibrous scarring in spinal cord injury of rat promotes long-distance regeneration of corticospinal tract axons, rescue of primary motoneurons in somatosensory cortex and significant functional recovery (Lewandowski and Steward, 2014)	European Journal of Neuroscience	137
51	Carmel J. B.	2010	Chronic electrical stimulation of the intact corticospinal system after unilateral injury restores skilled locomotor control and promotes spinal axon outgrowth (Carmel et al., 2010)	Journal of Neuroscience	135
52	Fuhrmann T.	2017	Combinatorial therapies after spinal cord injury: How can biomaterials help? (Führmann et al., 2017)	Advanced Healthcare Materials	134
53	Oudega M.	2012	Corticospinal reorganization after spinal cord injury (Oudega and Perez, 2012)	Journal of Physiology	134
54	Starkey M. L.	2005	Assessing behavioural function following a pyramidotomy lesion of the corticospinal tract in adult mice (Starkey et al., 2005)	Experimental Neurology	133
55	Lindau N. T.	2014	Rewiring of the corticospinal tract in the adult rat after unilateral stroke and anti-Nogo-A therapy (Lindau et al., 2014)	Brain	128
56	Buchli A. D.	2005	Inhibition of Nogo: a key strategy to increase regeneration, plasticity and functional recovery of the lesioned central nervous system (Buchli and Schwab, 2005)	Annals of Medicine	126
57	Rao J.-S.	2018	NT3-chitosan enables *de novo* regeneration and functional recovery in monkeys after spinal cord injury (Rao et al., 2018)	Proceedings of the National Academy of Sciences of the United States of America	125
58	Jin D.	2015	Restoration of skilled locomotion by sprouting corticospinal axons induced by co-deletion of PTEN and SOCS3 (Jin et al., 2015)	Nature Communications	125
59	Cafferty W. B. J.	2006	The Nogo–Nogo receptor pathway limits a spectrum of adult CNS axonal growth (Cafferty and Strittmatter, 2006)	Journal of Neuroscience	125
60	Dimou L.	2006	Nogo-A-deficient mice reveal strain-dependent differences in axonal regeneration (Dimou et al., 2006)	Journal of Neuroscience	125
61	Kim B.	2017	Can neurological biomarkers of brain impairment be used to predict poststroke motor recovery? A systematic review (Kim and Winstein, 2017)	Neurorehabilitation and Neural Repair	121
62	Lacroix S.	2004	Bilateral corticospinal projections arise from each motor cortex in the macaque monkey: a quantitative study (Lacroix et al., 2004)	Journal of Comparative Neurology	120
63	Griffin J. M.	2020	Therapeutic repair for spinal cord injury: combinatory approaches to address a multifaceted problem (Griffin and Bradke, 2020)	EMBO Molecular Medicine	118
64	Fry E. J.	2010	Corticospinal tract regeneration after spinal cord injury in receptor protein tyrosine phosphatase sigma deficient mice (Fry et al., 2010)	Glia	118
65	Meyers E. C.	2018	Vagus nerve stimulation enhances stable plasticity and generalization of stroke recovery (Meyers et al., 2018)	Stroke	117
66	Katoh H.	2019	Regeneration of spinal cord connectivity through stem cell transplantation and biomaterial scaffolds (Katoh et al., 2019)	Frontiers in Cellular Neuroscience	115
67	Du K.	2015	PTEN deletion promotes regrowth of corticospinal tract axons 1 year after spinal cord injury (Du et al., 2015)	Journal of Neuroscience	115
68	Fawcett J. W.	2015	The extracellular matrix in plasticity and regeneration after CNS injury and neurodegenerative disease (Fawcett, 2015)	Progress in Brain Research	115
69	Konishi J.	2005	MR tractography for the evaluation of functional recovery from lenticulostriate infarcts (Konishi et al., 2005)	Neurology	115
70	Rosenzweig E. S.	2009	Extensive spinal decussation and bilateral termination of cervical corticospinal projections in rhesus monkeys (Rosenzweig et al., 2009)	Journal of Comparative Neurology	114
71	Hollis E. R.	2009	Induction of corticospinal regeneration by lentiviral trkB-induced Erk activation (Hollis et al., 2009)	Proceedings of the National Academy of Sciences of the United States of America	112
72	Freund P.	2007	Anti-Nogo-A antibody treatment enhances sprouting of corticospinal axons rostral to a unilateral cervical spinal cord lesion in adult macaque monkey (Freund et al., 2007)	Journal of Comparative Neurology	110
73	Courtine G.	2005	Performance of locomotion and foot grasping following a unilateral thoracic corticospinal tract lesion in monkeys (*Macaca mulatta*) (Courtine et al., 2005)	Brain	109
74	Fouad K.	2004	Regenerating corticospinal fibers in the marmoset (*Callitrix jacchus*) after spinal cord lesion and treatment with the anti-Nogo-A antibody IN-1 (Fouad et al., 2004)	European Journal of Neuroscience	109
75	Wang Z.	2015	Overexpression of Sox11 promotes corticospinal tract regeneration after spinal injury while interfering with functional recovery (Wang et al., 2015)	Journal of Neuroscience	108
76	Han Q.	2020	Restoring cellular energetics promotes axonal regeneration and functional recovery after spinal cord injury (Han et al., 2020)	Cell Metabolism	107
77	Guggisberg A. G.	2019	Brain networks and their relevance for stroke rehabilitation (Guggisberg et al., 2019)	Clinical Neurophysiology	107
78	Ruitenberg M. J.	2005	NT-3 expression from engineered olfactory ensheathing glia promotes spinal sparing and regeneration (Ruitenberg, 2005)	Brain	105
79	Cho S.-H.	2007	Motor outcome according to the integrity of the corticospinal tract determined by diffusion tensor tractography in the early stage of corona radiata infarct (Cho et al., 2007)	Neuroscience Letters	104
80	Puig J.	2013	Decreased corticospinal tract fractional anisotropy predicts long-term motor outcome after stroke (Puig et al., 2013)	Stroke	102
81	Anderson K. D.	2005	Quantitative assessment of forelimb motor function after cervical spinal cord injury in rats: relationship to the corticospinal tract (Anderson et al., 2005)	Experimental Neurology	102
82	Brus-Ramer M.	2009	Motor cortex bilateral motor representation depends on subcortical and interhemispheric interactions (Brus-Ramer et al., 2009)	Journal of Neuroscience	101
83	Steward O.	2008	A re-assessment of the effects of a Nogo-66 receptor antagonist on regenerative growth of axons and locomotor recovery after spinal cord injury in mice (Steward et al., 2008)	Experimental Neurology	101
84	Moller M.	2007	Dynamic changes in corticospinal tracts after stroke detected by fibretracking (Moller et al., 2007)	Journal of Neurology Neurosurgery and Psychiatry	98
85	Stinear C. M.	2017	Proportional motor recovery after stroke: implications for trial design (Stinear et al., 2017)	Stroke	97
86	Freund P.	2009	Anti-Nogo-A antibody treatment promotes recovery of manual dexterity after unilateral cervical lesion in adult primates—re-examination and extension of behavioral data (Freund et al., 2009)	European Journal of Neuroscience	97
87	Sasaki M.	2006	Protection of corticospinal tract neurons after dorsal spinal cord transection and engraftment of olfactory ensheathing cells (Sasaki et al., 2006)	Glia	95
88	Fabes J.	2007	Regeneration-enhancing effects of EphA4 blocking peptide following corticospinal tract injury in adult rat spinal cord (Fabes et al., 2007)	European Journal of Neuroscience	94
89	Hilton B. J.	2017	Can injured adult CNS axons regenerate by recapitulating development? (Hilton and Bradke, 2017)	Development	93
90	Puig J.	2017	Diffusion tensor imaging as a prognostic biomarker for motor recovery and rehabilitation after stroke (Puig et al., 2017)	Neuroradiology	92
91	Morita T.	2016	Intravenous infusion of mesenchymal stem cells promotes functional recovery in a model of chronic spinal cord injury (Morita et al., 2016)	Neuroscience	92
92	Ueno M.	2012	Intraspinal rewiring of the corticospinal tract requires target-derived brain-derived neurotrophic factor and compensates lost function after brain injury (Ueno et al., 2012)	Brain	92
93	Liu Z.	2008	Contralesional axonal remodeling of the corticospinal system in adult rats after stroke and bone marrow stromal cell treatment (Liu et al., 2008)	Stroke	90
94	Danilov C. A.	2015	Conditional genetic deletion of PTEN after a spinal cord injury enhances regenerative growth of CST axons and motor function recovery in mice (Danilov and Steward, 2015)	Experimental Neurology	89
95	Auriat A. M.	2015	A review of transcranial magnetic stimulation and multimodal neuroimaging to characterize post-stroke neuroplasticity (Auriat et al., 2015)	Frontiers in Neurology	88
96	Liu J.	2015	Enhanced interhemispheric functional connectivity compensates for anatomical connection damages in subcortical stroke (Liu et al., 2015)	Stroke	87
97	Boato F.	2010	C3 peptide enhances recovery from spinal cord injury by improved regenerative growth of descending fiber tracts (Boato et al., 2010)	Journal of Cell Science	87
98	Zeng X.	2015	Integration of donor mesenchymal stem cell-derived neuron-like cells into host neural network after rat spinal cord transection (Zeng et al., 2015)	Biomaterials	84
99	Barthelemy D.	2010	Impaired transmission in the corticospinal tract and gait disability in spinal cord injured persons (Barthélemy et al., 2010)	Journal of Neurophysiology	84
100	Koch P.	2016	Structural connectivity analyses in motor recovery research after stroke (Koch et al., 2016)	Annals of Clinical and Translational Neurology	83

### 3.2 Analysis of the most productive countries

**Figure 3:**
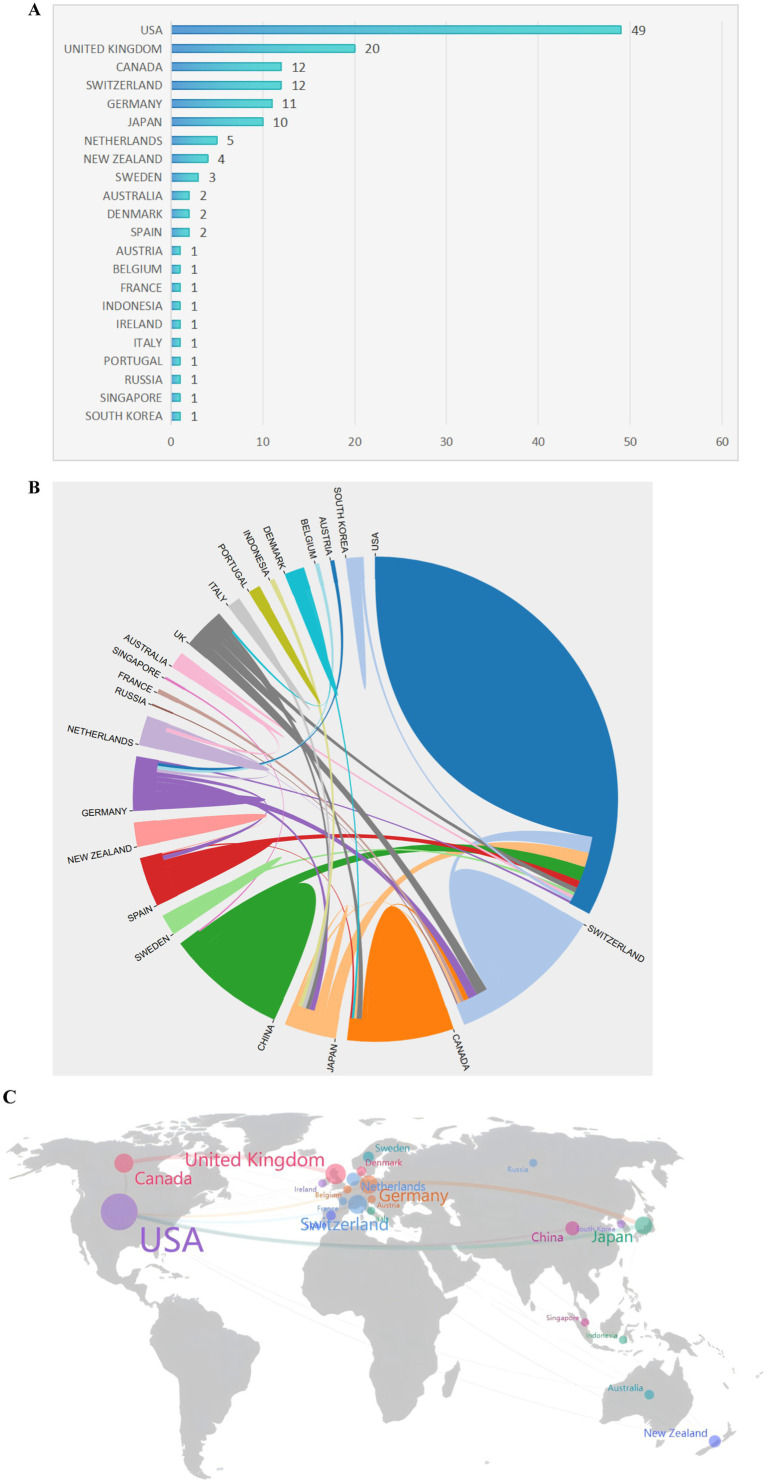
The most productive countries. **(A)** A network map showing the countries involved in this research area. **(B)** The number of publications by country. **(C)** A world map highlighting the most productive countries.

**Figure 4:**
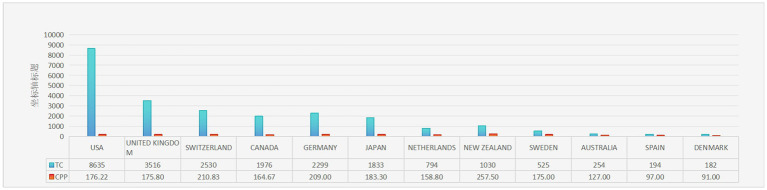
The 10 countries with the most publications. TC, total citations; CPP, citations per publication.

The contributions of 22 countries/regions to the top 100 most cited manuscripts are illustrated in Figures 3A,B. Eight of these countries each published more than three articles. Scimago Graphica software was used to graphically represent a world map in which countries with a significant presence among the top 100 cited publications are highlighted (Figure 3C). The sizes of the dots on the map correspond to the volume of articles contributed by each country, with lines indicating collaborative ties between nations. The United States led the field, accounting for 49 of the 100 papers and amassing 8,635 citations, which equates to an average of 176.22 citations per paper (Figure 4). The United Kingdom was second, with 20 papers and a total of 3,516 citations, averaging 175.80 citations per paper. Switzerland ranked third, with 12 papers and 2,530 citations, averaging 164.67 citations per paper. New Zealand had the distinction of having the highest average number of citations per publication, with a notable average of 257.50.

### 3.3 Institution analysis

**Figure 5:**
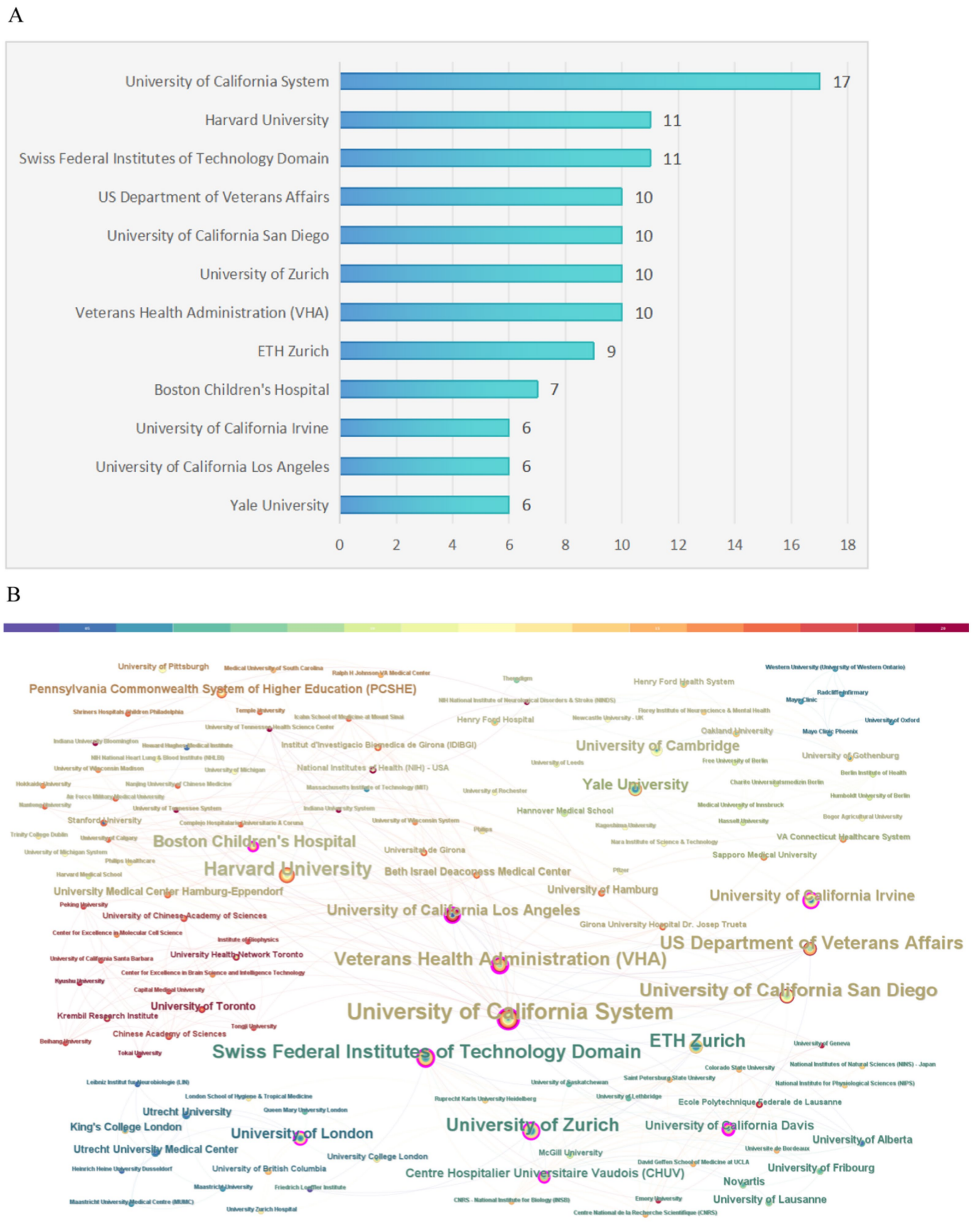
Institution analysis. **(A)** The most relevant institutions. **(B)** Partnerships among institutions.

Regarding institutional contributions, a total of 177 institutions were involved in producing the top 100 articles analyzed in this study. Figure 5A illustrates the number of publications for the most significant institutions. Notably, the University of California System stood out as the most prolific institution, contributing 17 of the most highly cited articles. CiteSpace software was employed to create a visual representation of the connections between these institutions, where the sizes of the nodes in the graph indicate each institution’s publication output, and lines represent collaborative efforts among them. The leading research institutions in terms of collaboration, including the University of California System, Harvard University, the Swiss Federal Institutes of Technology, the US Department of Veterans Affairs, UC San Diego, the University of Zurich, and the Veterans Health Administration, among others, are highlighted in Figure 5B.

### 3.4 Author analysis

**Figure 6:**
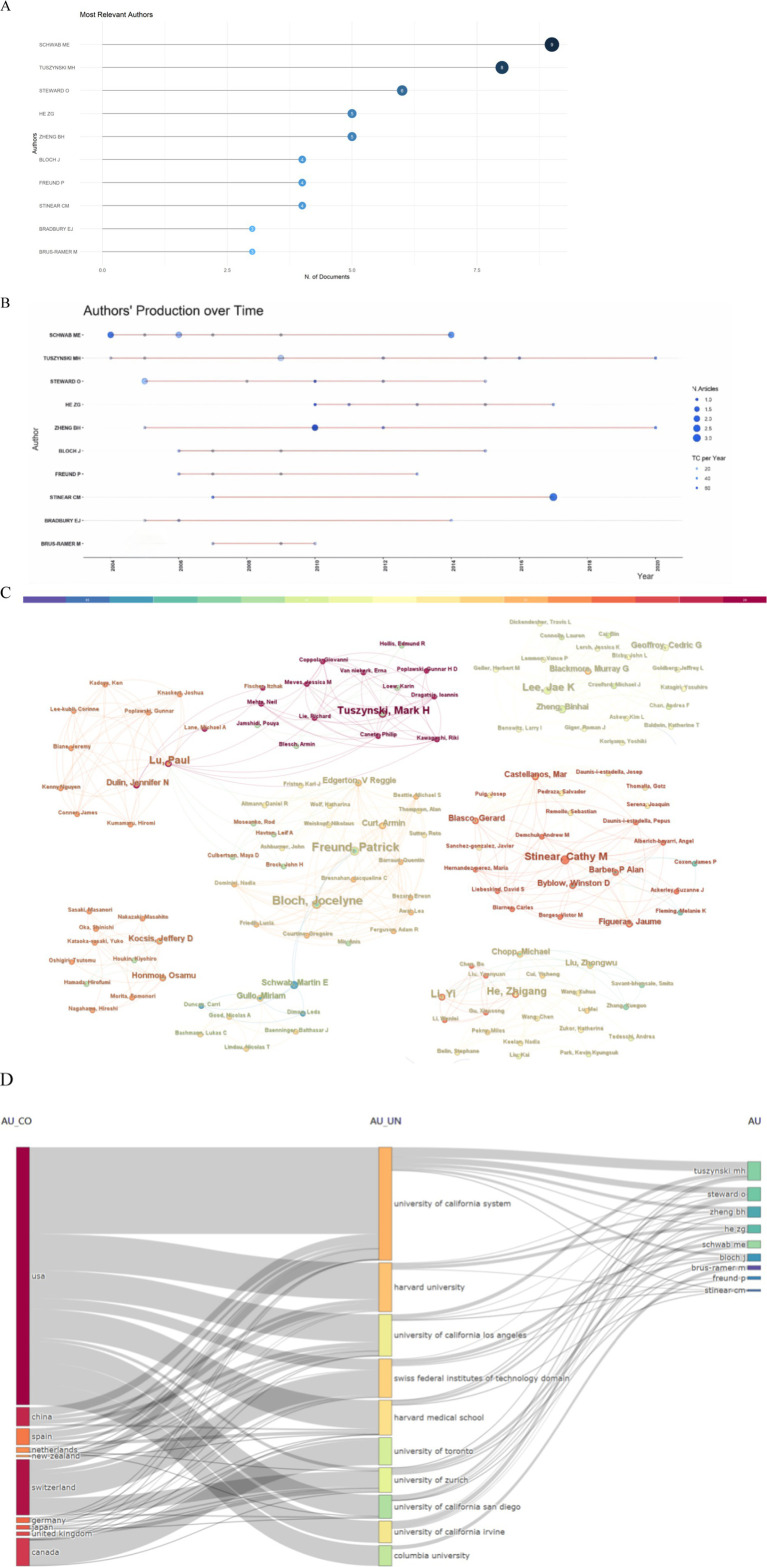
Author analysis. **(A)** The most relevant authors. **(B)** Article production over time. **(C)** A map of the collaborative relationship among authors. **(D)** Three-field plot (country-affiliation-author).

The Bibliometrix R package, a tool for visualizing and analyzing scientific literature data, was used to identify the most prominent authors in the field. As shown in Figure 6A, Schwab M. E., topped the list, with an impressive nine publications. A timeline of the publication history of the most prolific authors is presented in Figure 6B. A network where the size of each node is proportional to an author’s contribution to the top 100 articles, with lines indicating collaborative ties among them, is shown in Figure 6C. An analysis of this network revealed a high incidence of collaboration among authors, characterized by stable partnerships. Figure 6D provides an overview of the interconnectedness among countries, institutions, and authors, with a notable influence from USA-based authors.

### 3.5 Analysis of journals

Table 2 displays the relevant journals for the top 100 cited articles. The Journal of Neuroscience published the highest number of articles (*n* = 13), encompassing 2,300 citations. This was followed by Brain, which published 8 papers and accumulated 1,845 citations. Next was Experimental Neurology, which published 6 articles, obtaining 850 citations. Stroke also published 6 articles and received 730 citations, with an average citation count of 121.67. Furthermore, seven journals each published more than 3 articles—Proceedings of the National Academy of Sciences of the United States of America, European Journal of Neuroscience, and Nature Neuroscience, accumulating 807, 2,323, and 437 citations, respectively.

**Table 2:** Journals associated with the 100 most cited articles in the field of corticospinal tract regeneration (>1 paper).

Ranking	Journal	Documents	Total citations	IF in 2023	5 years IF	Average citations
1	Journal of Neuroscience	13	2,300	4.4	5.3	176.92
2	Brain	8	1,845	10.6	12.5	230.63
3	Experimental Neurology	6	850	4.6	4.8	141.67
3	Stroke	6	730	7.8	8.2	121.67
5	Proceedings of the National Academy of Sciences of the United States of America	5	807	9.4	10.8	161.40
6	Nature Neuroscience	4	2,323	21.2	25.6	580.75
6	European Journal of Neuroscience	4	437	2.7	3.2	109.25
8	Nature Medicine	3	680	58.7	59.2	226.67
8	Lancet Neurology	3	642	46.5	51.6	214.00
8	Neuron	3	618	14.7	16.9	206.00
8	Glia	3	362	5.4	6.6	120.67
8	Journal of Comparative Neurology	3	344	2.3	2.4	114.67
13	Nature Reviews Neuroscience	2	385	28.7	37.4	192.50
13	Journal of Neurophysiology	2	277	2.1	2.5	138.50

### 3.6 Keywords and research hot spots

**Figure 7:**
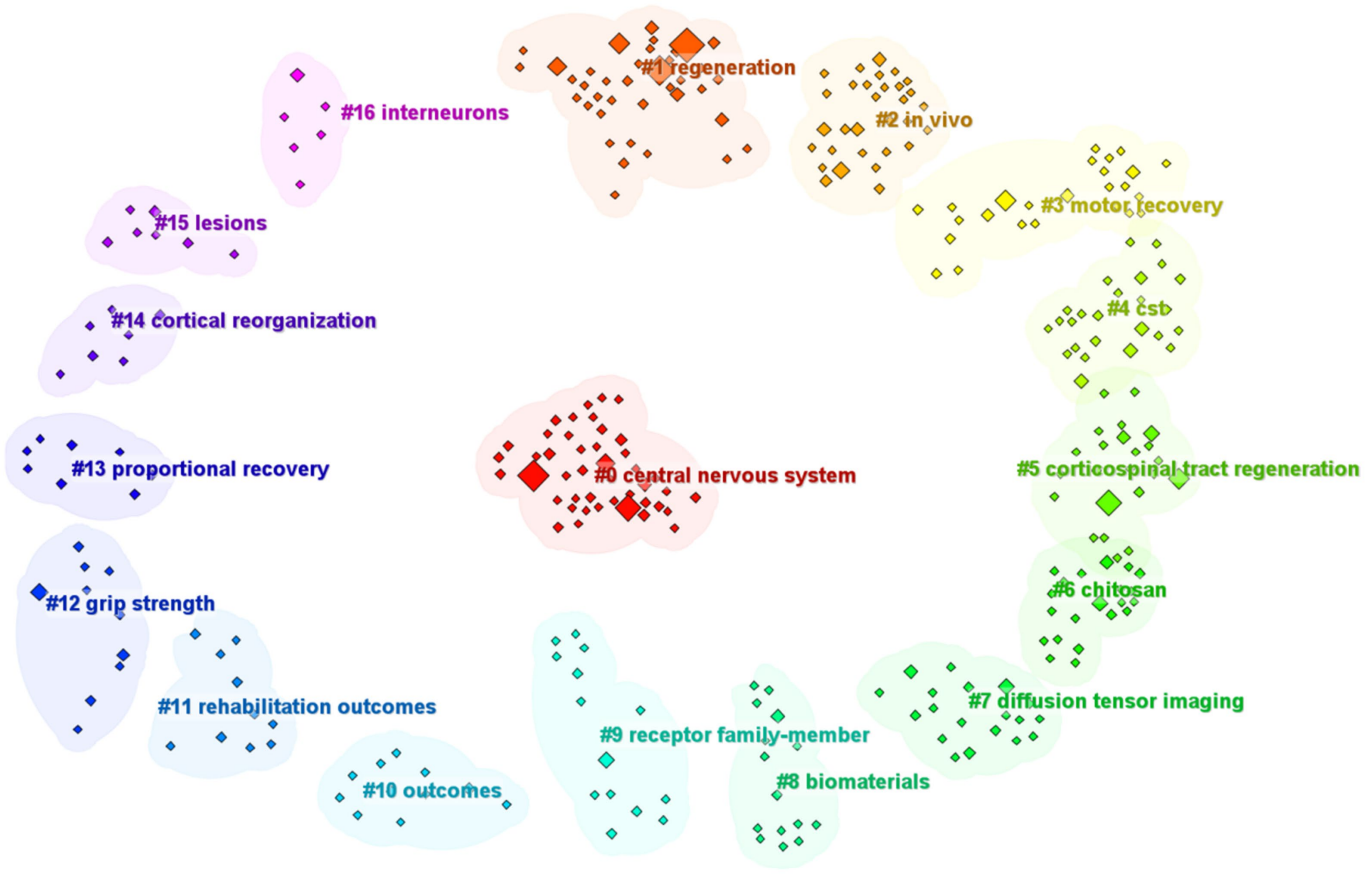
Keyword analysis: keyword cluster graph.

Keywords are essential for highlighting the core themes of a scholarly article, offering readers a clear understanding of the topic. The presence of two keywords in the same paper indicates a connection between them, with the frequency of their co-occurrence reflecting the strength of this link. By analyzing the patterns of keyword co-occurrence and identifying emerging trends, it is possible to identify trending subjects within an academic area over time. Keyword clustering can help to reveal the underlying structure of knowledge, enabling a more organized understanding of the domain within a specific field. Here, cluster analysis revealed that keywords within the field of CST regeneration could be partitioned into the following 16 categories (Figure 7): “central nervous system,” “regeneration,” “*in vivo*,” “motor recovery,” “CST,” “corticospinal tract regeneration,” “chitosan,” “diffusion tensor imaging,” “biomaterials,” “receptor family member,” “outcomes,” “rehabilitation outcomes,” “grip strength,” “proportional recovery,” “cortical reorganization,” “lesions,” and “interneurons.”

**Figure 8:**
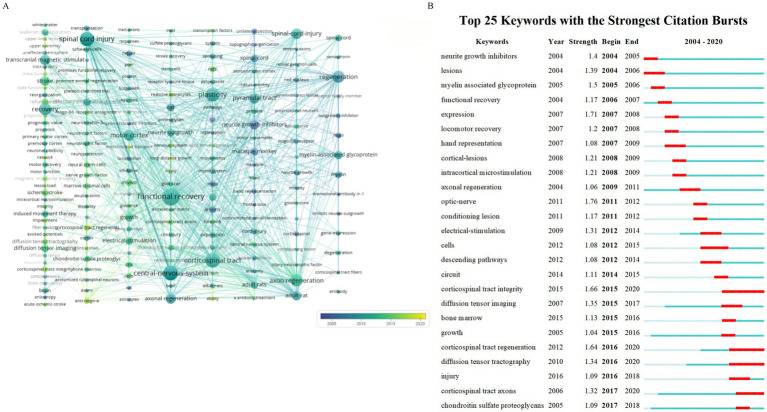
Keyword modifications over time. **(A)** Timeline view of the keywords. **(B)** Burst test of the keywords.

The chronological evolution of keyword usage patterns is shown in Figure 8. Meanwhile Figure 8A provides a visual representation of how the prominence of keywords fluctuates over time. The size of each block corresponds to the popularity of the keyword with larger blocks indicating more frequent usage. Keywords that have become more prevalent over recent years may become significant research areas in the future. Our analysis of keyword bursts pinpointed several noteworthy terms (Figure 8B) including “optic nerve,” “expression,” “corticospinal tract integrity,” “corticospinal tract regeneration,” “diffusion tensor tractography,” “myelin-associated glycoprotein,” “neurite growth inhibitors,” “electrical stimulation,” and “intracortical microstimulation.” Keywords with the strongest burst signals are indicative of current research frontiers within the field. Early bursting keywords suggest that initial research interest was concentrated in those areas while recent bursts indicate a sharp increase in interest in the topic. Figure 8B highlights the five keywords with the highest burst strengths namely “optic nerve,” “expression,” “corticospinal tract integrity,” “corticospinal tract regeneration,” and “diffusion tensor tractography,” with burst strengths of 1.76, 1.71, 1.66, 1.64, and 1.64 respectively. The first keywords to show a burst were “neurite growth inhibitors,” “lesions,” “myelin-associated glycoprotein,” “functional recovery,” “expression,” and “locomotor recovery,” representing early research emphasis. Meanwhile the most recent keywords to display a burst were “chondroitin sulfate proteoglycans,” reflecting a new area of intense research interest.

## 4 Discussion

In this study, we performed a comprehensive data and bibliometric analysis, focusing on the 100 most cited publications in the field of CST regeneration. This analytical strategy enabled a detailed examination of the evolution, key focus areas, and innovative trends in CST regeneration research, and offered valuable quantitative insights into both seminal and recent studies, thus deepening our understanding of the subject.

The top 100 publications accumulated a total of 18,321 citations, with citation counts ranging from 83 to 871 and a median of 136 citations per article. Schwab M. E. was identified as the most frequent contributor, having authored nine of these articles. The greatest number of papers, 49, originated from the United States, followed by the United Kingdom and Switzerland, each contributing 20 and 12 papers, respectively. The University of California System was the most prolific institution, producing 17 papers, followed by Harvard University with 11 papers and the Swiss Federal Institutes of Technology, also with 11 papers. Keyword analysis identified several areas of interest, including optic nerve, expression, corticospinal tract integrity, corticospinal tract regeneration, diffusion tensor tractography, myelin-associated glycoprotein, neurite growth inhibitors, electrical stimulation, and intracortical microstimulation. Further keyword analysis revealed the recent emergence of “chondroitin sulfate proteoglycans” as a prominent keyword in the field.

### 4.1 Optic-nerve

The optic nerve and the CST are distinct structures within the central nervous system (CNS), with each being responsible for different sensory and motor functions. The function of the optic nerve is to transmit visual signals from the retina to the brain, while the CST carries motor commands from the cerebral cortex to the spinal cord. Although the optic nerve and CST have separate functional roles, they share similar biological mechanisms and challenges in regard to regeneration (Gokoffski et al., 2020). Recent studies have revealed that the deletion of the SOCS3 gene facilitates the regeneration of the optic nerve. Moreover, the simultaneous knockout of the PTEN and SOCS3 genes has been reported to induce sustained axonal regeneration (Liu et al., 2011). These findings suggest that by modulating intrinsic signaling pathways, it is possible to promote the regeneration of axons within the CNS, including the optic nerve (Sun et al., 2011). These discoveries hold significant implications for understanding the mechanisms of regeneration in nerves such as the optic nerve and the CST and may pave the way for the development of novel neuroregenerative therapies.

### 4.2 Diffusion tensor tractography

Diffusion tensor tractography (DTT), leveraging data from diffusion tensor imaging (DTI), is a sophisticated neuroimaging technique designed to reconstruct and visualize the trajectories of white matter fibers within the CNS (Fortin et al., 2017), including the CST. This advanced methodology plays an integral role in the investigation of CST regeneration following SCI, facilitating the meticulous delineation of white matter fibers, thereby enabling a comprehensive understanding of the status of compromised neural pathways. Post-SCI, DTT serves not only to illustrate the damaged CST fibers but also to monitor their subsequent restoration (Feng et al., 2015; Cho et al., 2007). Juxtaposing pre-and post-injury DTT imagery allows to effectively gauge the efficacy of regenerative therapies (Guggisberg et al., 2019) as well as assess whether interventions such as neurotrophic factor administration, stem cell transplantation, or bio-scaffolding have indeed fostered CST regeneration (Puig et al., 2017). In summary, DTT stands as a robust instrument for the study and surveillance of CST regeneration, contributing substantially to advancements in the field of SCI treatment.

### 4.3 Myelin associated glycoprotein

Myelin-associated glycoprotein (MAG) is a crucial component of the myelin sheath in the CNS, playing a significant role in the development and functionality of the nervous system. MAG is highly abundant on myelinating oligodendrocytes within the CNS and is known to interact with specific neuronal gangliosides, such as GT1b and GD1a (Schnaar and Lopez, 2009), thereby contributing to the maintenance of the myelin-axon interface. However, MAG is also recognized as one of the classic myelin-associated inhibitors that suppress axonal regeneration following CNS injury, including damage to the CST (Harel and Strittmatter, 2006). The inhibitory effect of MAG on neurite outgrowth is mediated through its interaction with axonal receptors (Zheng et al., 2005), which leads to the collapse of axonal growth cones in a sialic acid binding-dependent manner. Furthermore, MAG signaling operates bidirectionally, engaging in both axon-to-myelin and myelin-to-axon communication. It has been demonstrated that MAG plays a pivotal role in the regulation of myelin formation and integrity, with its cytoplasmic domain binding to the cytoplasmic non-receptor tyrosine kinase Fyn, which is essential for initiating the myelination process. Understanding the role of MAG in the inhibition of axonal regeneration is vital for developing strategies aimed at promoting CST regeneration and functional recovery following SCI. Targeting MAG or its signaling pathways may enhance the intrinsic capacity of the CNS for self-repair and remyelination, offering promising avenues for therapeutic intervention (Cafferty et al., 2010).

### 4.4 Electrical-stimulation

Electrostimulation, a therapeutic modality widely employed in clinical and research domains, holds substantial potential for facilitating functional recovery, particularly the regeneration of the CST, following CNS injury. The fundamental principle of this treatment involves the application of electrical currents to specific cortical areas to activate neuronal cells and elicit sensory or motor responses. In the context of CST regeneration, electrostimulation may contribute to the stimulation of the motor cortex, thereby promoting functional restoration (Lindau et al., 2014). Electrostimulation could also support the regeneration of the CST, by activating specific neural pathways that promote the reestablishment of connections between the brain and spinal cord. Research has demonstrated that electrostimulation can enhance axonal regeneration through various mechanisms (Liu et al., 2017); for instance, it can increase the expression of neurotrophic factors, thereby providing a conducive environment for axonal growth. Moreover, electrostimulation can augment axonal growth and regenerative capabilities by influencing intracellular signaling pathways. Clinical studies applying electrostimulation have also reported positive outcomes. For instance, it was demonstrated that electrostimulation can advance motor function recovery after SCI by enhancing the activity of residual neural pathways, leading to improved motor control and coordination. However, the efficacy of electrostimulation treatment may be contingent on factors such as the intensity, frequency, duration, and precision of targeting of the stimulation. To achieve optimal therapeutic outcomes, these parameters require meticulous adjustment and optimization. In summary, as a non-invasive or minimally invasive treatment approach, electrostimulation has demonstrated significant potential and value in the regeneration and functional recovery of the CST. With continued technological advancements and research progress, electrostimulation is set to become a vital treatment strategy for CNS injuries, particularly the regeneration of the CST.

### 4.5 Intracortical microstimulation

Intracortical microstimulation (ICMS) is a neuroscientific technique that involves the application of small electrical currents to specific areas of the cerebral cortex through implanted microelectrodes. This method is used to activate neural cells and can elicit specific sensory or motor responses, depending on the area of the cortex being stimulated. ICMS has been used to generate motor maps, which are detailed representations of how different areas of the motor cortex control specific body movements. In the context of “CST regeneration,” ICMS could potentially play a role in rehabilitation by stimulating the motor cortex in a way that promotes functional recovery after CNS injury (Shelchkova et al., 2023). By activating specific neural pathways, ICMS might help to reestablish connections between the brain and the spinal cord, thereby aiding CST regeneration. In summary, ICMS represents a promising approach for the study and potential treatment of CNS injuries, particularly CST regeneration, as it enables the precise stimulation and modulation of neural activity.

### 4.6 Chondroitin sulfate proteoglycans

Chondroitin sulfate proteoglycans (CSPGs) play a pivotal role in the regeneration and plasticity of the CNS, participating in a multitude of biological processes throughout development, adulthood, and aging. As integral components of the extracellular matrix, CSPGs interact with a variety of growth-active molecules, thereby influencing neural regeneration and plasticity. CSPGs are often considered to be inhibitors of CST regeneration, given their propensity to accumulate at sites of CNS injury, where they form glial scars that impede axonal regeneration. It has been demonstrated that the degradation of CSPGs mediated by enzymes such as chondroitinase ABC can enhance axonal regeneration and facilitate functional recovery (Barritt et al., 2006). Moreover, CSPGs are involved in modulating immune cell responses, thereby impacting the pathogenesis of chronic inflammation and demyelinating diseases. Following SCI, the digestion of CSPGs can mitigate proinflammatory responses within the inflammatory environment and promote the clearance or reduce the recruitment of microglia and peripheral myeloid cells at the lesion site, thereby contributing to the resolution of the inflammatory response. Regarding therapeutic strategies, manipulating the expression of CSPGs or their signaling pathways can foster CST regeneration. For instance, inhibiting the synthesis of CSPGs can diminish their inhibitory effects within the CNS (García-Alías et al., 2009), offering potential therapeutic approaches for the treatment of CNS injuries, stroke, and neurodegenerative diseases. These observations highlight the multifaceted role of CSPGs in CST regeneration, encompassing the inhibition of axonal regeneration, the modulation of immune responses, and the promotion of the resolution of inflammation. Investigating the role of CSPGs in CST regeneration can provide vital information for the development of novel therapeutic strategies for CST injuries.

### 4.7 Clinical translation

The CST is vital for CNS function and enhancing its regeneration following SCI is critical for motor function recovery. Clinically, a spectrum of strategies is being pursued to stimulate CST neuron regeneration and establish functional networks post-SCI. These include the use of growth factors, cell therapies, and genetic manipulation to boost the inherent ability of CST neurons to regrow and myelinate. Neuromodulation techniques such as transcranial magnetic stimulation and transcranial direct current stimulation aim to adjust neural activity and support CNS plasticity, thereby aiding motor recovery after SCI. The development of implantable devices for continuous spinal cord stimulation represents an active research area, intended to foster an environment that is beneficial for CST regeneration. Biomaterial scaffolds that span spinal cord lesions are also being engineered, providing a substrate for axonal regrowth and offering nourishment to support axonal regeneration, potentially also in the CST. Combinatorial therapeutic approaches are often required for SCI given its multifaceted nature, involving the merging of neuromodulation with biomaterials, drug treatments, and physical rehabilitation to optimize CST regeneration and functional restoration. Translating CST regeneration research into clinical practice is a dynamic and progressive discipline. Achieving this necessitates a cross-disciplinary strategy, integrating knowledge from neuroscience, bioengineering, and rehabilitation medicine to develop therapies that can significantly enhance the quality of life of patients with SCI.

## 5 Limitations

This research has several limitations. While the Web of Science is a prominent database for literature retrieval, it does not include all existing publications. To improve the precision of our analysis, we opted for a topic-based search approach rather than relying on subject headings. Although this method yielded accurate results, there is potential for broader coverage. Relying on citation frequency as a criterion for selection might result in the exclusion of recently published works that may be influential but have not yet amassed a substantial number of citations. Moreover, there is a possibility of citation bias, as articles from renowned institutions or prominent authors might garner more citations than equally meritorious ones from less recognized origins.

## 6 Conclusion

This investigation presents a bibliometric analysis of CST regeneration, underscoring the pivotal role of the United States as a leader in this scientific domain. Our findings unveiled the dynamics and trends within the field of CST regeneration, providing a scientific foundation for clinical applications. Our analysis indicated that the application of CST regeneration in the clinic should be optimized through interdisciplinary collaboration, enabling the exploration and validation of a variety of therapeutic approaches, including the use of neurotrophic factors, stem cell therapies, biomaterials, and electrical stimulation. Concurrently, additional clinical trials are necessary to test the safety and efficacy of these therapeutic methods and develop assessment tools for monitoring the recovery of patients. Furthermore, rehabilitation strategies should be refined, while professional education and training should be provided to enhance the understanding of CST regeneration treatments among both medical professionals and patients. International cooperation is also crucial, fostering the sharing of data and experiences to advance global research. Securing policy and funding support will aid in driving research progress and therapeutic innovation. Lastly, establishing long-term tracking systems is crucial for evaluating the long-term outcomes and safety of CST regeneration treatments. The implementation of these strategies promises to enhance therapeutic outcomes and the quality of life of patients with SCI.
